# Detection of Central Nervous System Infiltration by Myeloid and Lymphoid Hematologic Neoplasms Using Flow Cytometry Analysis: Diagnostic Accuracy Study

**DOI:** 10.3389/fmed.2018.00070

**Published:** 2018-05-23

**Authors:** Laiz Cameirão Bento, Rodolfo Patussi Correia, Anderson Marega Alexandre, Sonia Tsukasa Nosawa, Eduardo de Carvalho Pedro, Andressa da Costa Vaz, Daniela Schimidell, Gustavo Bruniera Peres Fernandes, Carlos Augusto Senne Duarte, Rodrigo de Souza Barroso, Nydia Strachman Bacal

**Affiliations:** ^1^Clinical Pathology Laboratory, Division of Hematology and Flow Cytometry, Hospital Israelita Albert Einstein, São Paulo, Brazil; ^2^Senne Liquor Laboratório, São Paulo, Brazil; ^3^Hematology Laboratory, Centro de Hematologia de São Paulo, São Paulo, Brazil

**Keywords:** flow cytometry, cerebrospinal fluid, cytology, central nervous system, neoplasms

## Abstract

**Introduction:**

Infiltration of the central nervous system (CNS) by hematologic or lymphoid malignant cells can cause extensive neurological damage, be progressive and fatal. However, usually, the cerebrospinal fluid (CSF) has low cellularity and rapid cell degeneration, which can impair cytometry analysis. Storage and transport measures, sample preparation, and staining protocols can interfere with diagnostic accuracy.

**Objective:**

To calculate the diagnostic performance of flow cytometry (FC) using a cell stabilizer for sample preservation compared to cytomorphology in the detection of CNS infiltration by lymphoid and hematologic neoplasms.

**Methods:**

Cell samples from all consecutive patients with suspected infiltration by hematological malignancies evaluated between January 2014 and December 2016 were included. Cases were analyzed by FC using a cell preservation medium and cytomorphology. Sensitivity and specificity were calculated.

**Results:**

From 414 CSF samples, 72 had a phenotype compatible with characteristics of infiltration by hematological disease, whereas cytology was positive for 35 cases. FC showed higher sensitivity and specificity when compared to cytomorphology, particularly in cases with cellularity under 5 leukocytes/mm^3^.

**Conclusion:**

We demonstrated that collecting CSF in a medium that preserves the stability of the sample improves accuracy when compared to cytomorphology, particularly in low-volume and low-cellularity samples.

## Introduction

The involvement of the central nervous system (CNS) by hematologic malignancies is considered a major complication in patients with B- or T-cell lymphomas and in patients with acute lymphoid or myeloid leukemia (1). Neoplastic cell infiltration into the CNS may cause extensive neurological damage and can be progressive and fatal if it is not detected in time (2, 3). Meningeal infiltration by neoplastic cells occurs in approximately 5–15% of patients with leukemia and lymphoma (4). The involvement of the CNS can occur through dissemination of neoplastic cells through arteries, perineural and perivascular spaces, and infiltration of malignant cells from solid tumors (4–6).

Currently, the main tools used for detection of CNS infiltration by neoplastic cells include neurological clinical assessment, neuroimaging studies, and cytomorphology (7). Symptoms of CNS infiltration may be present in several conditions other than hematologic malignancies, the clinical manifestations are late and usually occur in advanced stages of disease (7). Some neuroimaging techniques, such as magnetic resonance imaging, are sensitive but have low specificity (8). Cytological examination of the cerebrospinal fluid (CSF) is considered a gold standard technique; however, it has low sensitivity with false positive results ranging from 20 to 60%, especially in low-cellularity samples (9, 10), which can delay diagnosis and the correct treatment of patients (7, 11).

Laboratory techniques with greater accuracy and sensitivity include molecular biology, molecular genetics, and flow cytometry (FC) (7, 12). FC complements cytology as a technique to test the CSF (3, 13–15). Cytometry can enhance the possibility of diagnosing lymphomas in the CSF, because it is able to detect abnormal cells in samples containing only 0.9% of monoclonal B-lymphocytes (2, 16, 17). Currently, in patients with clinical suspicion of CNS infiltration by neoplastic cells, cytology, and immunophenotyping by FC are the techniques recommended by the National Comprehensive Cancer Network (18).

Recent advances have shown that the use of multiparameter FC, such as 10-parameter single-tube detection, has considerably increased the specificity and sensitivity in detecting cells with abnormal phenotypes (1, 14, 15, 17, 19). Nonetheless, in most cases, the CSF has low cellularity with rapid cell degeneration. Therefore, recent studies have suggested some storage and transport guidelines, as well as sample preparation and staining protocols, to improve the quality of results (1).

### Objective

The purpose of this diagnostic accuracy study is to evaluate the diagnostic performance of FC using a cell stabilizer for sample preservation compared to cytomorphology in the detection of CNS infiltration by lymphoid and hematologic neoplasms.

## Materials and Methods

### Study Design, Participants, and Ethics

This is a diagnostic accuracy study of FC results compared to cytomorphology as the gold standard technique, in the diagnosis of CNS infiltration by neoplastic cells. Participants were all patients who had their tissue samples tested by FC between January 2014 and December 2016 in the same institution. To be included in this study, patients should have clinical suspicion or be under follow up for hematological malignancy, or they suffered from neurological conditions, such as encephalitis, epilepsy, seizures, among others (the study of the CSF was performed to rule out any hypothesis of oncohematological disease). All patients underwent examination of CSF cells by FC.

The Institutional Review Board reviewed the study protocol and approved the use of data collected for this study. The human tissue and fluid samples analyzed were obtained from patients routinely subjected to immunophenotyping in a clinical FC laboratory for diagnosis of hematologic neoplasms, irrespective of this study. The study did not offer any direct benefits to patients or interfere with patient diagnosis or treatment.

### Procedures

In order to preserve and prolong cell viability, the samples submitted for immunophenotyping were collected in cell stabilizer Transfix, in a tube containing the anticoagulant ethylenediaminetetracetic acid (EDTA), observing the ratio of 1:10 Tranfix/CSF and a volume ranging between 3 and 5 ml.

In cases of suspected lymphoproliferative disease and other clinical conditions, the staining strategy initially used for immunophenotyping was the Euroflow Small Sample Tube, with the antibodies shown in Table 1. Patients with suspected infiltration by acute myeloid leukemia (AML), multiple myeloma, and acute B- and T-cell lymphoid leukemia were stained according to Table 2. All antibodies were standardized, titrated, and validated by our laboratory.

**Table 1:** Antibodies used for staining in the cases of suspicion of lymphoproliferative disease.

Fluorochrome	DLPC	Clone	Manufacturer
FITC	CD8^+^ lambda	UCHT-4/polyclonal	Cytognos
PE	CD56^+^ kappa	N901/polyclonal	Cytognos
PERCP-Cy5.5	CD4	SK3	Becton Dickinson
PC7	CD19	J3-119	Beckman Coulter
APC	CD3^+^ CD14	SK7/MφP9	Becton Dickinson
APC-H7	CD38	HB7	Becton Dickinson
V450	CD20	L27	Becton Dickinson
V500	CD45	2D1	Becton Dickinson

*FITC, fluorescein isothiocyanate; PE, phycoerythrin; PERCP-Cy5.5, phycoerythrin and cyanine dye; PC7, phycoerythrin-Cy7; APC, allophycocyanin; APC-H7, allophycocyanin H7*.

**Table 2:** Antibodies used to investigate infiltration of hematologic malignancy in the central nervous system.

Fluorochrome	B-ALL	Clone	AML	Clone	T-ALL	Clone	MM	Clone	Manufacturer
FITC	CD38	T16	^a^	–	CD8	UCHT-4	CD38	T16	Cytognos
PE	CD56	N901	CD56	N901	CD4	N901	CD56	N901	Cytognos
PERCP-Cy5.5	CD34	8G12	CD34	8G12	CD34	8G12	^a^	–	BD
PC7	CD19	J3-119	CD117	104D2D1	CD19	J3-119	CD19	J3-119	BC
APC	CD3^+^ CD14	SK7/MφP9	CD3^+^ CD14	SK7/MφP9	CD3^+^ CD14	SK7/MφP9	CD3^+^ CD14	SK7/MφP9	BD
APC-H7	CD10	HB7	CD38	HB7	CD38	HB7	^a^	–	BD
V450	CD20	L27	CD20	L27	CD3cy	UCHT1	CD45	2D1	BD
V500	CD45	2D1	CD45	2D1	CD45	2D1	CD138	MI15	BD

*FITC, fluorescein isothiocyanate; PE, phycoerythrin; PERCP-Cy5.5, phycoerythrin and cyanine dye; PC7, phycoerythrin-Cy7; APC, allophycocyanin; APC-H7, allophycocyanin H7; BC, Beckman Couter; BD, Becton Dickinson; AML, acute myeloid leukemia; T-ALL, T-cell lymphoblastic leukemia; B-ALL, B-cell lymphoblastic leukemia*.

*^a^Empty spaces that can be filled in by previously markers known upon diagnosis and that can help investigate infiltration*.

### Cytomorphology and FC

The cell count was performed approximately 30 min after puncture using a Fuchs-Rosenthal chamber and sample cellularity ranged between 0 and 908 cells/μl.

For slides preparation, we used cytocentrifugation at 1,000 rpm, for 5 min, using 200 μl of fresh CSF samples. Samples were stained according to the Leishmann method. Each case was interpreted by an experienced cytomorphologist who was unaware of the results of FC. Samples were considered negative in the absence of morphologically anomalous population. The results were considered positive when there was the presence of cells with atypical characteristics, such as high nucleus cytoplasm and presence of nucleolus.

According to the standard technical procedure, the cell samples were then centrifuged, now at a speed of 1,100 rpm, for 10 min, washed with 10 ml of phosphate buffer saline (PBS) + 2% fetal bovine serum (FBS). For clinical suspicion of infiltration of mature B-cell neoplasms, the washing procedure was repeated to eliminate proteins, which could interfere in detection of clonality with kappa and lambda light chain, and/or prevent unspecific staining.

The cell pellet was then stained with fluorochrome conjugates of monoclonal antibodies, and incubated for 20 min, at room temperature, and protected from light. Red blood cell lysis was performed (whenever necessary) with an ammonium chloride buffer for 5 min; cells were washed with 3 ml PBS + 2% FBS and resuspended in 0.5 ml PBS. Finally, the stained samples were immediately acquired in the Becton Dickinson FACS Canto II flow cytometer. Data analysis was performed using the Infinicyt software (Cytognos).

### Statistical Analysis

Cytomorphology and FC results were compared using absolute and relative frequencies, and the agreement between methods was verified using kappa and diagnostic measures (sensitivity and specificity) (20). Cytomorphology was considered the gold standard technique. Statistical analyses were performed with SPSS, version 22.0 for Windows (SPSS Inc., Chicago, IL, USA).

## Results

Between January 2014 and December 2016, 414 CSF samples (239, or 57.7% were men) were analyzed by FC and cytomorphology to investigate for CNS infiltration by hematological malignancy. The mean age was 48.5 years (range of 2–90 years).

### Patients

Of the patients, 306 were clinically suspected of or being followed up for hematological malignancy, and 108 had other clinical conditions, such as encephalitis, epilepsy, seizures, among others. Of the 414 samples tested, 342 showed no immunophenotypic changes suggestive of CNS infiltration by hematological malignancies. Even in 335 samples who had low cellularity (<5 cells/μl) defined by the Fuchs-Rosenthal global chamber count, FC was able to detect T-, B-, natural killer (NK)-lymphocytes, and monocytes (Table 3).

**Table 3:** Frequency of population detected by flow cytometry.

Cerebrospinal fluid (CSF) cell count	Number of cases	Frequency of population in CSF			
		CD3	CD14	CD3(−)CD56(+)	CD19
0	104	96.9	83.6	50.0	7.0
1 cells/μl	143	99.3	94.4	73.4	23.8
2 cells/μl	35	100.0	100.0	88.6	37.1
3 cells/μl	18	100.0	100.0	83.3	61.1
4–5 cells/μl	11	100.0	100.0	100.0	45.5

### Negative FC Results

**Figure 1:**
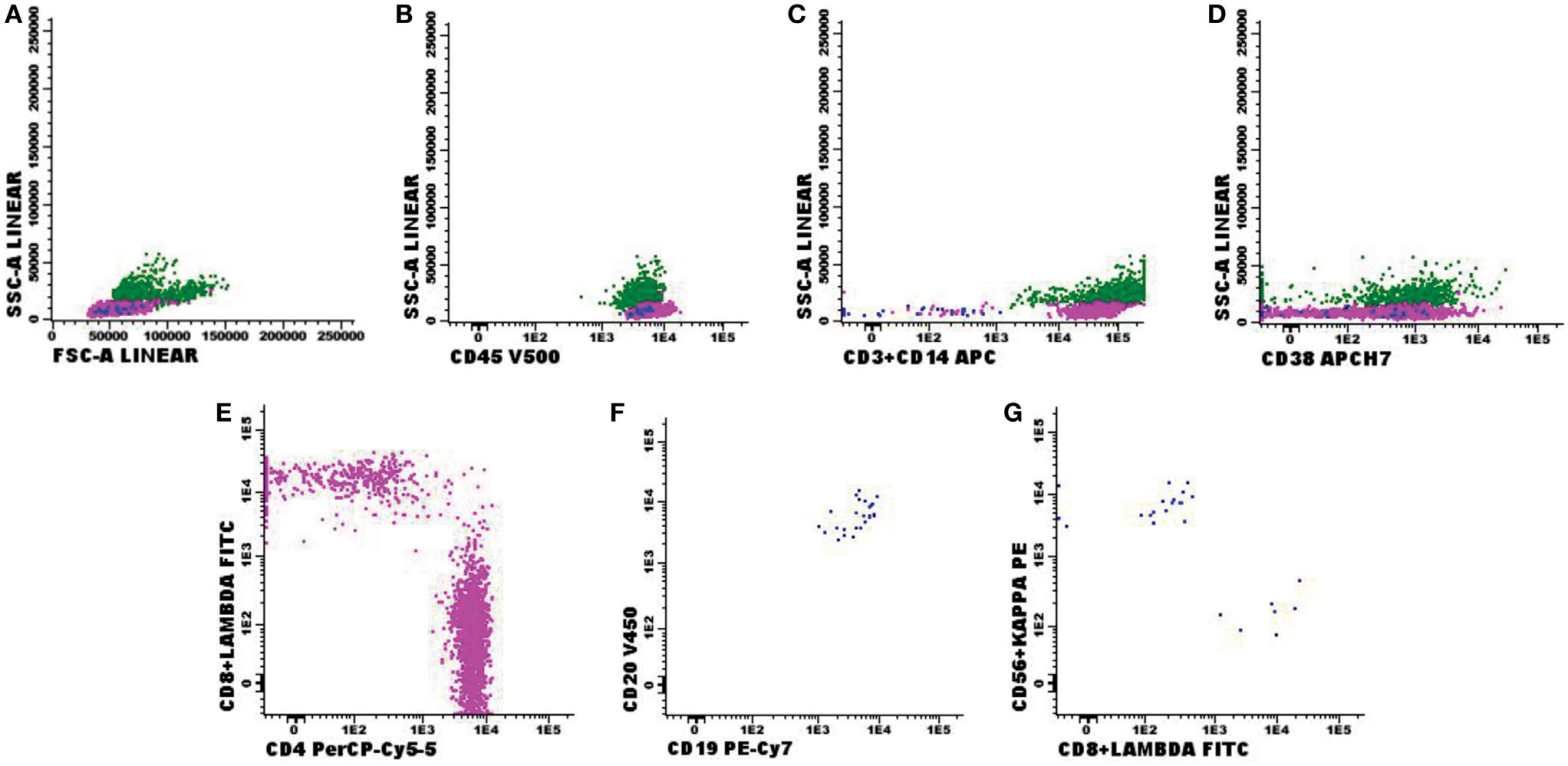
Negative flow cytometry result for suspected central nervous fluid (CSF) infiltration by hematological malignancies. Presumptive diagnosis: central nervous system disorder. **(A,B)** Normal forward-scattered light (FSC size) and side-scattered light (complexity—SSC); **(C–E)** normal antigenic expression of CD3 in T-lymphocytes, CD4 and CD8 expression in CD3^+^CD4^+^ and CD3^+^CD8^+^ lymphocyte subtypes, CD14 expression in monocytes and CD38 expression and CD56^+^CD3^−^ natural killer (NK) cells; **(F,G)** polyclonal CD19^+^CD20^+^ B-lymphoid population for kappa (73%) and lambda (27%) light chains. Sample with 3 leukocytes/mm^3^ and 1 red blood cell/mm^3^, acquisition of 8,425 events in the FACS Canto II (Becton Dickinson), and analysis performed with the software Infinicyt (Cytognos). The frequency of populations in the viable cell region and CD45^+^ (3,385 events) was 68.6% CD3 T-lymphocytes—in that, 52.6% CD4 T-lymphocytes and 13.9% CD8 T-lymphocytes; 0.9% NK cells; 0.7% mature and polyclonal B-lymphocytes (kappa/lambda ratio: 2.38); and 29.8% monocyte lineage cells.

Comparing the 342 negative FC samples (Figure 1) with the morphological samples, 304 were also negative in the cytomorphological test. Only 2 samples showed positive cytomorphology for neoplastic cells, while 9 had inconclusive results in the same test. Morphological testing was not possible in 3 samples due to low cellularity, and 24 samples were missing an order for chemical and cytological analyses.

### Positive FC Results

**Figure 2:**
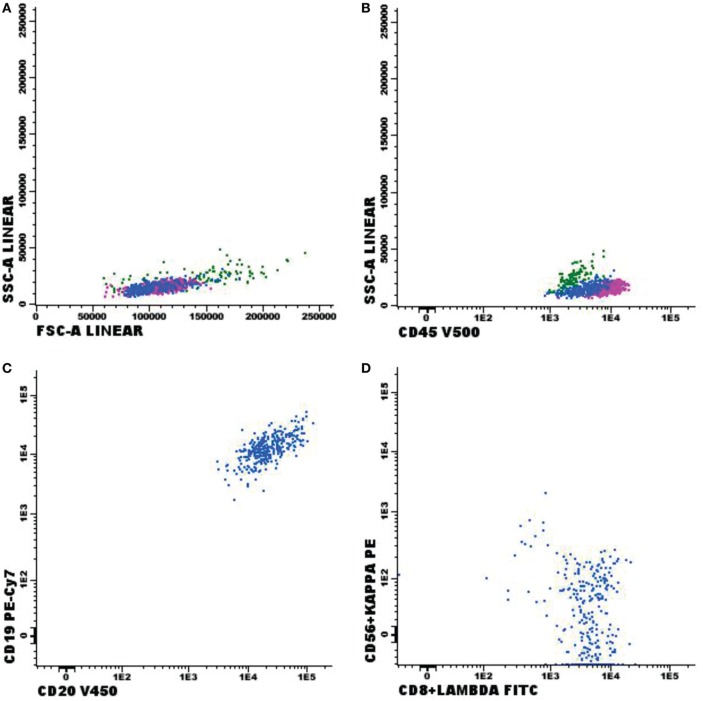
Flow cytometry results of suspected central nervous system infiltration by B-cell non-Hodgkin lymphoma. Presumptive diagnosis: B-cell non-Hodgkin lymphoma. **(A,B)** Normal forward-scattered light (FSC size) and side-scattered light (complexity—SSC); **(C)** mature CD19^+^ and CD20^+^ B-lymphoid population; **(D)** mature monoclonal lambda-light-chain-expressing B-lymphoid population. Sample collected in Transfix^®^/ethylenediaminetetracetic acid with an approximate volume of 5 ml and 2 leukocytes/mm^3^ and 0 red blood cells/mm^3^. Samples with 8,575 events acquired in Canto II (Becton Dickinson) and analyzed using the Infinicyt software (Cytognos). Frequency of populations in the viable cell region and CD45^+^ cell (1,000 events).

**Figure 3:**
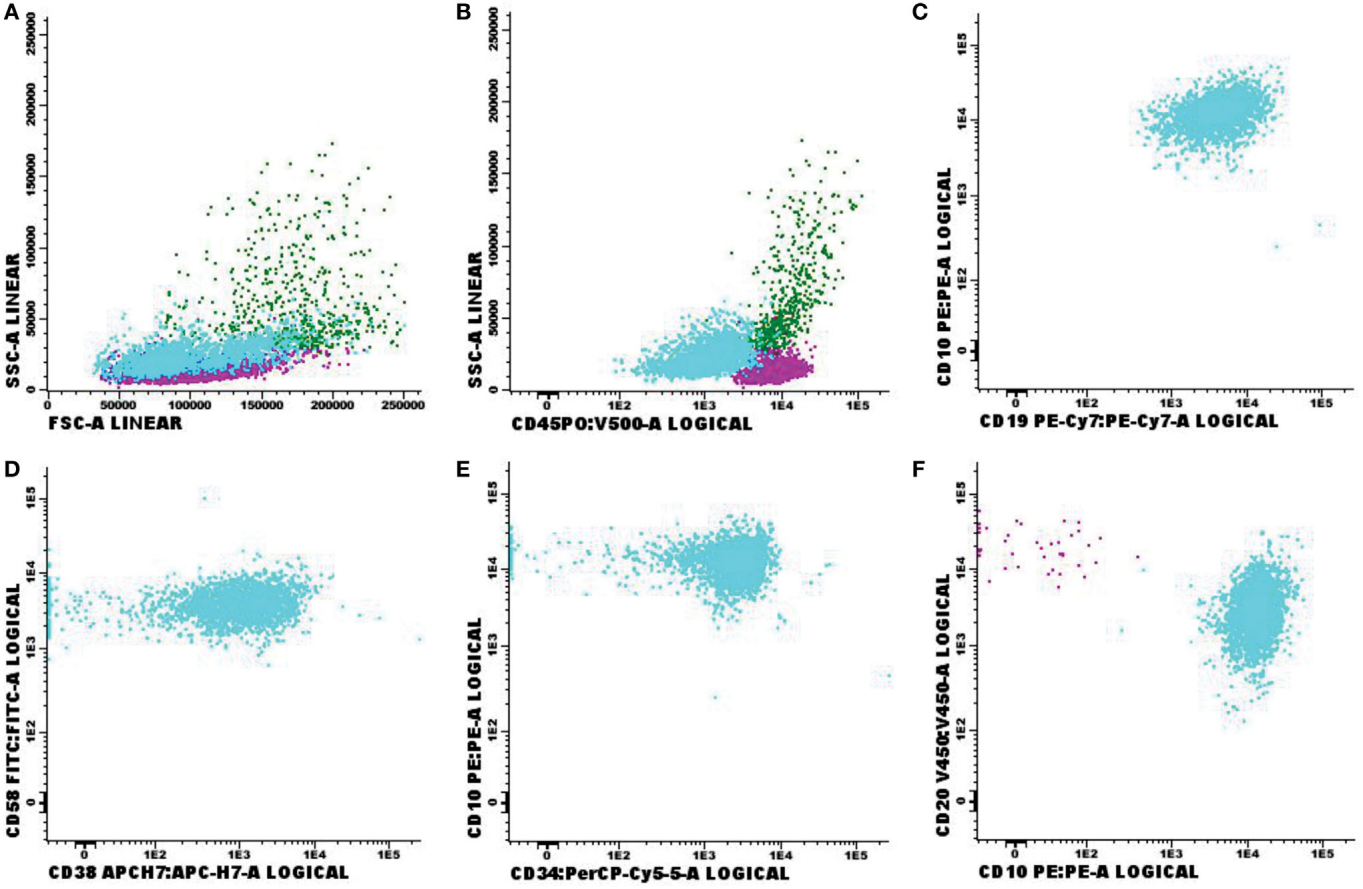
Flow cytometry results of suspected central nervous system infiltration by B-cell acute lymphoblastic leukemia. Presumptive diagnosis: suspected B-cell lymphoblastic leukemia. **(A)** Forward-scattered light (FSC size) and side-scattered light (complexity—SSC); **(B)** low-complexity blast population weakly expressing CD45 (light blue); **(C)** population of CD19^+^ and CD10^+^ B-lymphoid blasts; **(D)** strong expression of CD58 and moderate expression of CD38 in blast population; **(E)** CD10^+^ and CD34^+^ expression; **(F)** strong expression of CD10 in blast population. Sample collected in Transfix^®^/ethylenediaminetetracetic acid with an approximate volume of 5 ml and 3 leukocytes/mm^3^ and 0 red blood cells/mm^3^. Samples with 20,904 events acquired in Canto II (Becton Dickinson) and analyzed using the Infinicyt software (Cytognos). The frequency of populations in the viable cell region and CD45^+^ was 14,210 events.

**Figure 4:**
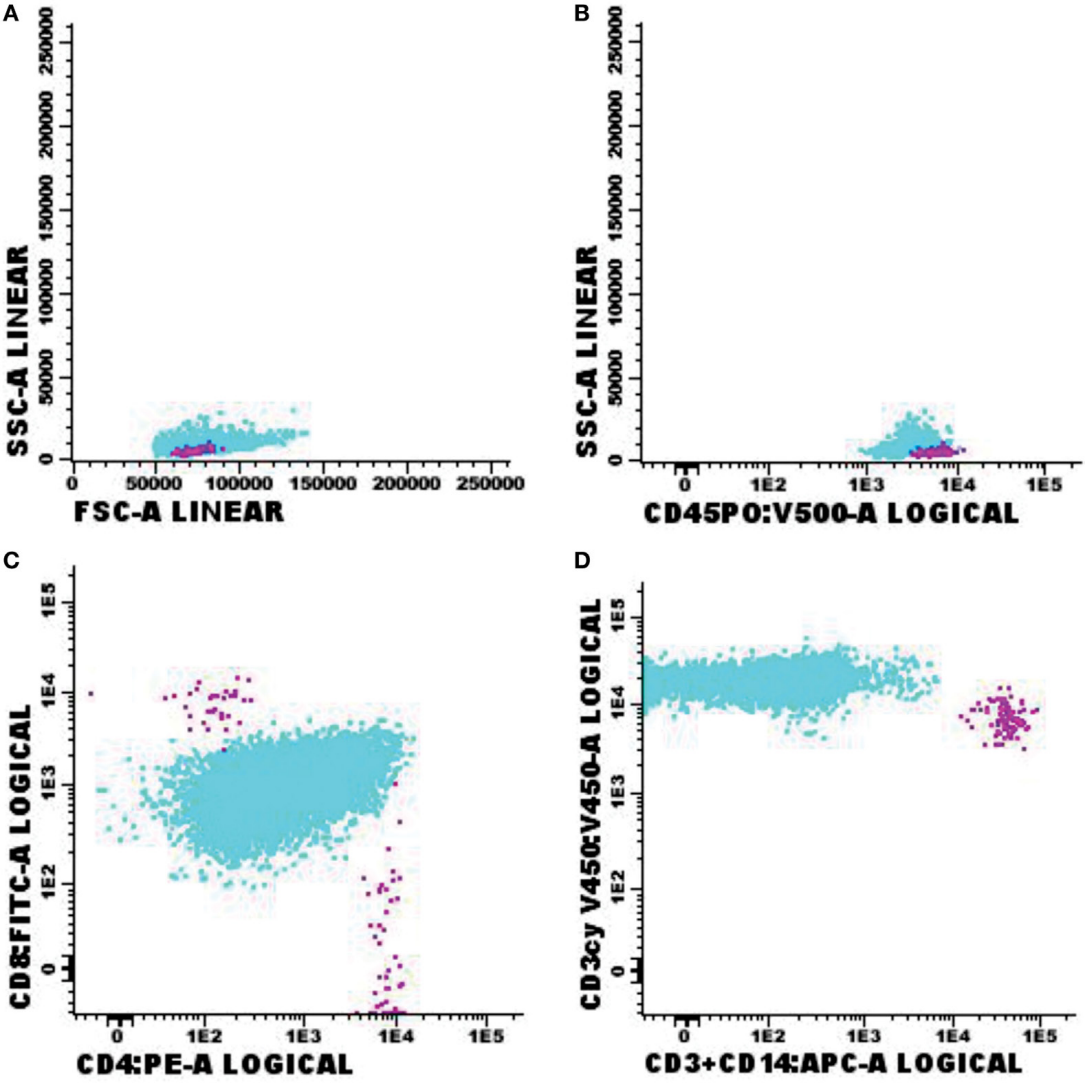
Flow cytometry results of suspected central nervous system infiltration by T-cell acute lymphoblastic leukemia. Presumptive diagnosis: T-cell lymphoblastic leukemia. **(A)** Forward-scattered light (FSC size) and side-scattered light (complexity—SSC); **(B)** low-complexity blast population (light blue) moderately/strongly expressing CD45; **(C)** CD4/CD8 double positive blast population; **(D)** intracytoplasmic CD3-positive and surface CD3-negative blasts. Sample collected in Transfix/ethylenediaminetetracetic acid with an approximate volume of 5 ml and 3 leukocytes/mm^3^ and 10 red blood cells/mm^3^. Samples with 11,575 events acquired in Canto II (Becton Dickinson) and analyzed using the Infinicyt software (Cytognos). The frequency of populations in the viable cell region and CD45^+^ cell was 7,913 events.

**Figure 5:**
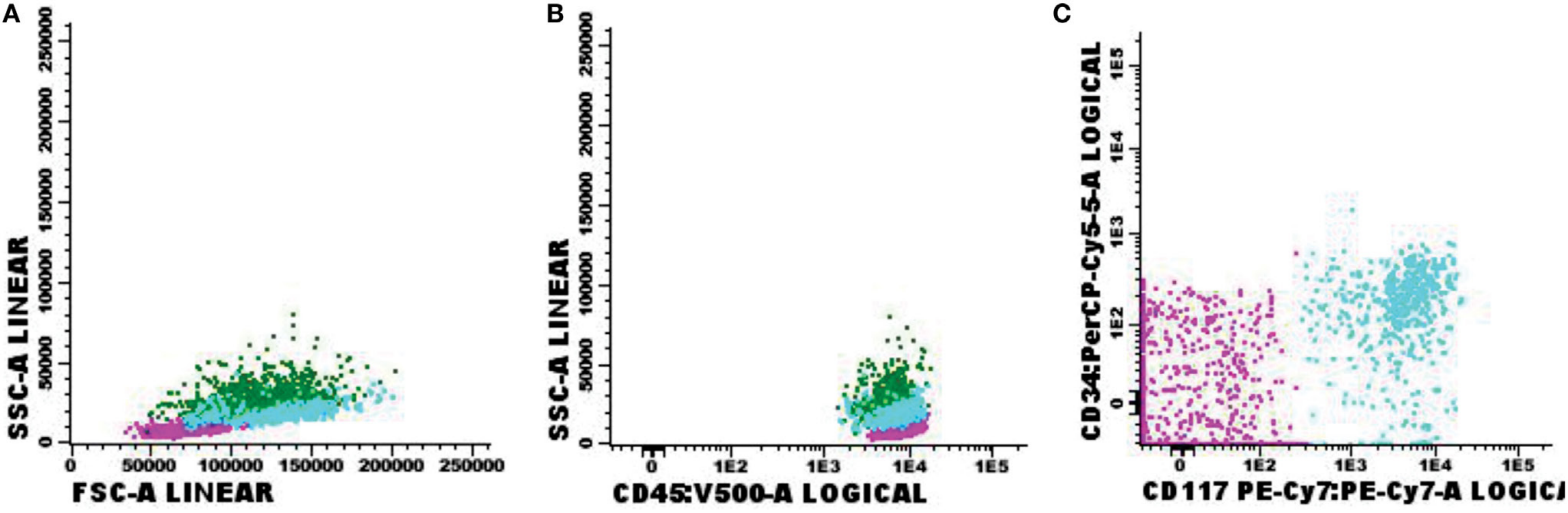
Flow cytometry results of suspected central nervous system infiltration by immature myeloid population. Presumptive diagnosis: acute myeloid leukemia. **(A)** Forward-scattered light (FSC size) and side-scattered light (complexity—SSC); **(B)** low-complexity blast population (light blue) strongly expressing CD45; **(C)** blast population with abnormal CD117 expression and absent CD34 expression. Sample collected in Transfix/ethylenediaminetetracetic acid with an approximate volume of 5 ml and 1 leukocyte/mm^3^ and 2 red blood cells/mm^3^. Samples with 6,375 events acquired in Canto II (Becton Dickinson) and analyzed using the Infinicyt software (Cytognos). The frequency of populations in the viable cell region and CD45^+^ was 2,541 events.

Immunophenotypic abnormalities suggestive of CNS infiltration by hematological malignancies were found in 72 cases studied. Among the positive cases, 33 samples were classified as non-Hodgkin B-cell lymphoma (B-NHL, Figure 2); 10 as B-cell lymphoblastic leukemia (Figure 3); 1 blastic plasmacytoid dendritic cell neoplasm; 9 with T-cell lymphoblastic leukemia (Figure 4); 2 T/NK-cell lymphoblastic leukemia; 4 blastic plasmacytoid dendritic cell neoplasms, and 13 showed abnormal immature myeloid population (AML) (Figure 5).

**Figure 6:**
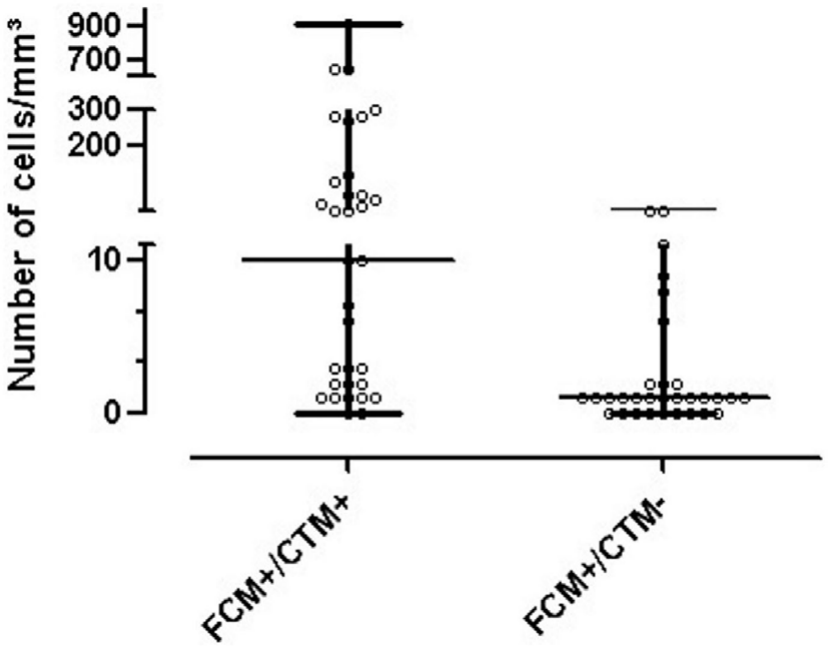
Positive results for flow cytometry and results found for cytomorphology.

In respect to the 72 positive FC samples, 35 were also positive on the cytomorphology test, 2 were inconclusive, 29 were negative, 1 sample was not processed due to low cellularity, and in 5 cases, there were no samples available because the attending physician did not fill in the request for chemical and cytological testing. Interestingly, the CNS infiltration by hematological malignancies was identified even in samples with <5 cells/μl that represented 55.6% (40/72) of positive FC tests (*p* < 0.001, Figure 6). Furthermore, in these 40 positive FC samples, 57.5% (23/40) had negative cytomorphology tests.

**Figure 7:**
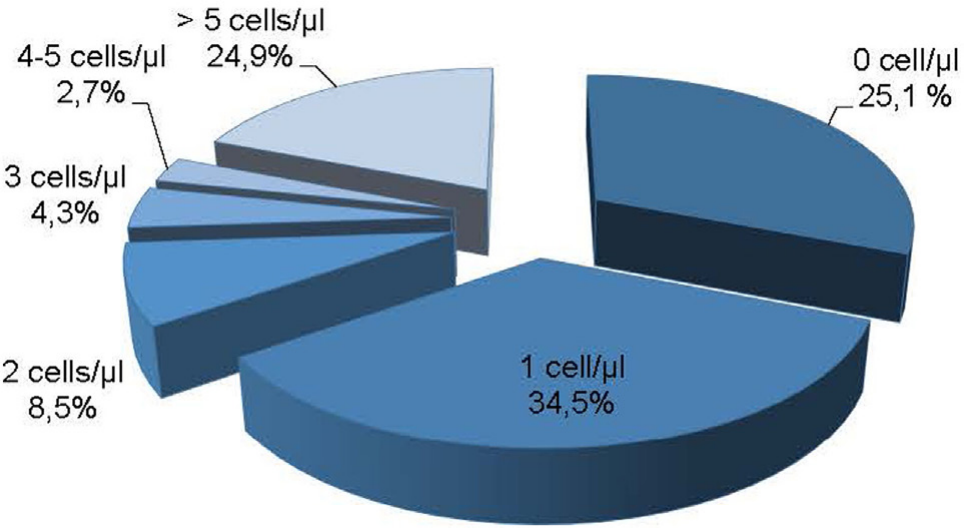
Samples grouped according to cellularity.

In the study of 414 cases, 370 presented positive or negative results for FC and cytomorphology. FC showed higher sensitivity and specificity for 370 samples when compared to cytomorphology, particularly in cases with cellularity under 5 leukocytes/mm^3^ (Table 4). This scenario of low cellularity represented 75.1% of the samples analyzed (311/414) (Figure 7).

**Table 4:** FC versus cytomorphology in the detection of infiltration of the central nervous system by hematologic malignancy.

		Cytomorphology								
Leukocyte count	FC	Negative		Positive		Total		Kappa	Sens.	Spec.

		*n*	%	*n*	%	*n*	%	(IC: 95%)	(IC: 95%)	(IC: 95%)
≤5 mm^3^	Negative	274	88.1	0	0.0	274	88.1	0.518	100	92.3
	Positive	23	7.4	14	4.5	37	11.9	(0.351; 0.685)	(76.8; 100)	(88.6; 95)
	Total	297	95.5	14	4.5	311	100			

>5 mm^3^	Negative	30	50.8	2	3.4	32	54.2	0.724	91.3	83.3
	Positive	6	10.2	21	35.6	27	45.8	(0.548; 0.9)	(72; 98.9)	(67.2; 93.6)
	Total	36	61.0	23	39.0	59	100			

Total	Negative	304	82.2	2	0.5	306	82.7	0.649	94.6	91.3
	Positive	29	7.8	35	9.5	64	17.3	(0.537; 0.761)	(81.8; 99.3)	(87.7; 94.1)
	Total	333	90.0	37	10.0	370	100			

*FC, flow cytometry; Sens., sensitivity; Spec., specificity*.

## Discussion

This study reports the technical improvements made to increase test accuracy, sample stability, and compare CSF analysis by FC and cytomorphology. The technical procedures of FC have been recently improved aiming to increase test reliability and make better use of samples. Our study showed that 72 samples had a phenotype compatible with infiltration by hematologic disease, whereas cytology was positive in 35 cases. Thus, FC was much more accurate and sensitive when compared with cytomorphology, primarily in low-cellularity and low-volume samples.

The investigation of CSF infiltration by hematologic neoplasms by conventional morphologic study has low sensitivity even when associated with neuroimaging studies, as well as neurological signs and symptoms. Despite the high specificity of cytomorphology, the literature reports false negative results range between 20 and 60% (2, 16, 21). Currently, conventional cytology tests yield positive results for neoplastic cells at concentrations starting at 5% (22). However, the cytomorphological analysis may be impaired because of the paucity of cells in CSF. Moreover, the identification of neoplastic cells in CSF can be a challenge due to similarities between benign and malignant cells. Several studies have demonstrated a significant increase in sensitivity of meningeal infiltration detection in patients with hematologic malignancies when the samples are evaluated by FC comparing the cytomorphology, ratifying the importance of immunophenotyping in CSF analysis (21, 23–25).

Due to higher sensitivity, FC is important to detect occult or subclinical CNS involvement during maintenance therapy and clinical follow-up, especially when cytomorphology is negative. The detection of subclinical CNS involvement is associated with poor prognosis and a higher risk of relapse in acute lymphoblastic leukemia and B-cell lymphomas (2, 16, 24, 26).

However, FC still has some limitations for CSF testing due to low cellularity, aggravated by small samples and poor cell viability. In addition to these limitations, peripheral blood contamination could alter results, and it is very difficult to identify. Thus, CSF immunophenotyping requires specific technical procedures to be correctly conducted (1). In our data, 6 samples of 72 positive cases by FC were suggestive of contamination, and despite this, the ability to detect neoplastic cells in CSF by FC is greater than cytomorphology regardless of whether the cells are primary of the CNS or the result from peripheral blood contamination by puncture trauma. We know that this possibility of contamination is aggravated at the time of diagnosis of acute leukemias, when the neoplastic cells circulating in the peripheral blood may be in greater numbers. However, in the central neural system post-treatment evaluations of acute leukemias, the presence of circulating neoplastic cells is rare, which may indicate that the immunophenotypic findings suggest CNS infiltration and not contamination of the material at this clinical time.

In our study, FC was able to identify no neoplastic CSF cells and infiltrating neoplastic cells even in samples with low-cellularity and low-volume that represented 75.1% of our samples (Figure 7). Interestingly, even in CSF with no cells defined by Fuchs-Rosenthal chamber count, FCM was able to detect T-, B-, NK-lymphocytes, and monocytes (Table 3). As expected, the detection capability is proportional to CSF cells concentration. Our data showed that in a CSF with 2 cells/μl all T-cells and monocytes can be identified, and for NK-, B-lymphocytes the cellularity requirement varies between 3 and 5 cells/μl.

Altogether, these results support previous observations that FC is more sensitive than cytomorphology (2, 21), and that FC was able to detect CSF disease more frequently than cytomorphology (16). In this study, FC could detect CSF involvement in 11 samples while cytomorphology was able to detect only 1 case. The explanation to the higher sensitivity is that cytomorphology shows positive or suspicious results only in samples with increased cellularity. All this progress has been possible due to technical standardization for better cell concentration in a single tube, and updating of our immunophenotyping protocol, from 4 to 8 colors and 13 parameters. A better cell concentration provided satisfactory results even in samples with low cellularity and the change in immunophenotyping protocol allowed the staining in a single tube, increasing sensibility. The use of analysis software, such as Infinicyt, enabled a more accurate and reproducible data analysis, removing interfering factors, such as doublets, degenerated cells, and unspecific staining. Moreover, the use of the cell stabilizer Transfix allowed the receipt of samples from distant centers, avoiding stability problems. However, the cell stabilizer cannot be used for morphological analysis.

One of the major challenges faced by laboratories in CSF processing is rapid cell degeneration in samples. Thirty minutes after collection, the cell count starts to decrease significantly (27). The use of collection media is recommended to solve this problem (1). The two media used by most laboratories are the Roswell Park Memorial Institute medium and Transfix (Cytomarl, UK).

Transfix is a stabilizer that prevents cell loss and reduces autofluorescence. Some studies showed that using Transfix in CSF samples prevents cell loss and allows the sample to be stored for 18–72 h until it is processed (1, 27). Moreover, the complexity features of lymphocytes are maintained, cell autofluorescence decreases, and cell fixation does not interfere with monoclonal antibody staining. However, there is a decrease in the size and complexity of granulocyte and monocyte populations in case of prolonged storage in Transfix, which could hinder cytomorphological testing (27). FC is a powerful and sensitive technique for testing CSF with suspected infiltration of lymphoma or leukemia. With all these improvements, it was possible to increase the accuracy and sensitivity of the detection of B- and T-lymphocytes, and their subtypes (CD3^+^, CD3^+^CD4^+^, and CD3^+^CD8^+^), as well as NK cells and monocytes, even in samples with low cellularity and low volume, and both in non-neoplastic and neoplastic samples.

Our data corroborate the literature findings and allow us to conclude that FC is a highly sensitive tool for detection of hematologic neoplasms cells in CSF, especially in low-level CNS involvement. This information could be useful for close monitoring of patients, changes in therapy strategy, and identify patients with high risk of relapse.

## Conclusion

In this study, we demonstrated that the CSF can be used for disease detection and screening of major leukocyte populations, even in low-volume and low-cellularity samples. This is possible if the sample is collected in a medium that preserves sample stability, immunophenotyping is performed with technical procedures specific for CSF samples, if appropriate immunophenotyping panels and a flow cytometer with 8-color/13-parameter configuration are used, as well as modern data analysis software.

## Ethics Statement

The Institutional Review Board reviewed the study protocol and approved the use of data collected for this study. The human tissue and fluid samples analyzed were obtained from patients routinely subjected to immunophenotyping in a clinical flow cytometry laboratory for diagnosis of hematologic neoplasms, irrespective of this study. The study did not offer any direct benefits to patients or interfere with patient diagnosis or treatment. For blinding purposes, the ethics committee name was removed from the submitted text. However, the IRB that evaluated the study is Hospital Israelita Albert Einstein (HIAE), in São Paulo, Brazil.

## Author Contributions

LB participated in the study concept, collected and analyzed data, created the figures, wrote the article, and reviewed the final version. RC collected and analyzed data, participated in manuscript writing, and reviewed the final version. AA participated in statistical analyses, interpreted data and participated in the figure creation, and then reviewed the final version of the manuscript. AV, DS, EP, and SN contributed equally in flux cytometry data collection and analysis and reviewed the final version of the manuscript. RB, CD, and GF participated in data interpretation, correlating laboratorial and clinical data, reviewed the figures and the flow cytometry data, and reviewed the final version of the manuscript. NB participated in the study concept and in data interpretation, correlating laboratorial and clinical data, and reviewed the final version to be published.

## Conflict of Interest Statement

The authors declare that the research was conducted in the absence of any commercial or financial relationships that could be construed as a potential conflict of interest.

## References

[1] Kraan J Gratama JW Haioun C Orfao A Plonquet A Porwit A Flow cytometric immunophenotyping of cerebrospinal fluid. Curr Protoc Cytom (2008) Chapter 6:Unit 6.25. 10.1002/0471142956.cy0625s45 18770650 PMC10790684

[2] Bromberg JE Breems DA Kraan J Bikker G van der Holt B Smitt PS CSF flow cytometry greatly improves diagnostic accuracy in CNS hematologic malignancies. Neurology (2007) 68(20):1674–9. 10.1212/01.wnl.0000261909.28915.83 17502548

[3] Pittman M Treese S Chen L Frater JL Nguyen TT Hassan A Utility of flow cytometry of cerebrospinal fluid as a screening tool in the diagnosis of central nervous system lymphoma. Arch Pathol Lab Med (2013) 137(11):1610–8. 10.5858/arpa.2012-0313-OA 24168498

[4] Chamberlain MC Carcinomatous meningitis. Arch Neurol (1997) 54(1):16–7. 10.1001/archneur.1997.00550130008003 9006407

[5] Gonzalez-Vitale JC Garcia-Bunuel R. Meningeal carcinomatosis. Cancer (1976) 37(6):2906–11. 10.1002/1097-0142(197606)37:6<2906::AID-CNCR2820370648>3.0.CO;2-D 949711

[6] Grossman SA Krabak MJ. Leptomeningeal carcinomatosis. Cancer Treat Rev (1999) 25(2):103–19. 10.1053/ctrv.1999.0119 10395835

[7] Weston CL Glantz MJ Connor JR. Detection of cancer cells in the cerebrospinal fluid: current methods and future directions. Fluids Barriers CNS (2011) 8(1):14. 10.1186/2045-8118-8-14 21371327 PMC3059292

[8] Straathof CS de Bruin HG Dippel DW Vecht CJ. The diagnostic accuracy of magnetic resonance imaging and cerebrospinal fluid cytology in leptomeningeal metastasis. J Neurol (1999) 246(9):810–4. 10.1007/s004150050459 10525979

[9] Colocci N Glantz M Recht L. Prevention and treatment of central nervous system involvement by non-Hodgkin’s lymphoma: a review of the literature. Semin Neurol (2004) 24(4):395–404. 10.1055/s-2004-861534 15637651

[10] Pui CH Thiel E. Central nervous system disease in hematologic malignancies: historical perspective and practical applications. Semin Oncol (2009) 36(4 Suppl 2):S2–16. 10.1053/j.seminoncol.2009.05.002 19660680 PMC2805279

[11] Clarke JL Perez HR Jacks LM Panageas KS Deangelis LM. Leptomeningeal metastases in the MRI era. Neurology (2010) 74(18):1449–54. 10.1212/WNL.0b013e3181dc1a69 20439847 PMC2871005

[12] Shalaby T Achini F Grotzer MA Targeting cerebrospinal fluid for discovery of brain cancer biomarkers. J Cancer Metastasis Treat (2016) 2:176–87. 10.20517/2394-4722.2016.12

[13] Nückel H Novotny JR Noppeney R Savidou I Dührsen U. Detection of malignant haematopoietic cells in the cerebrospinal fluid by conventional cytology and flow cytometry. Clin Lab Haematol (2006) 28(1):22–9. 10.1111/j.1365-2257.2006.00741.x 16430456

[14] Subira D Castañón S Aceituno E Hernández J Jiménez-Garófano C Jiménez A Flow cytometric analysis of cerebrospinal fluid samples and its usefulness in routine clinical practice. Am J Clin Pathol (2002) 117(6):952–8. 10.1309/123P-CE6V-WYAK-BB1F 12047148

[15] Craig FE Ohori NP Gorrill TS Swerdlow SH. Flow cytometric immunophenotyping of cerebrospinal fluid specimens. Am J Clin Pathol (2011) 135(1):22–34. 10.1309/AJCPANA7ER1ABMZI 21173121

[16] Hegde U Filie A Little RF Janik JE Grant N Steinberg SM High incidence of occult leptomeningeal disease detected by flow cytometry in newly diagnosed aggressive B-cell lymphomas at risk for central nervous system involvement: the role of flow cytometry versus cytology. Blood (2005) 105(2):496–502. 10.1182/blood-2004-05-1982 15358629

[17] Finn WG Peterson LC James C Goolsby CL. Enhanced detection of malignant lymphoma in cerebrospinal fluid by multiparameter flow cytometry. Am J Clin Pathol (1998) 110(3):341–6. 10.1093/ajcp/110.3.341 9728609

[18] Brem SS Bierman PJ Brem H Butowski N Chamberlain MC Chiocca EA Central nervous system cancers. J Natl Compr Canc Netw (2011) 9(4):352–400. 10.6004/jnccn.2011.0036 21464144

[19] French CA Dorfman DM Shaheen G Cibas ES. Diagnosing lymphoproliferative disorders involving the cerebrospinal fluid: increased sensitivity using flow cytometric analysis. Diagn Cytopathol (2000) 23(6):369–74. 10.1002/1097-0339(200012)23:6<369::AID-DC1>3.0.CO;2-3 11074639

[20] Fleiss JL The Design and Analysis of Clinical Experiments. New York: Wiley (1986).

[21] Quijano S López A Manuel Sancho J Panizo C Debén G Castilla C Identification of leptomeningeal disease in aggressive B-cell non-Hodgkin’s lymphoma: improved sensitivity of flow cytometry. J Clin Oncol (2009) 27(9):1462–9. 10.1200/JCO.2008.17.7089 19224854

[22] Hollender A Kvaloy S Nome O Skovlund E Lote K Holte H. Central nervous system involvement following diagnosis of non-Hodgkin’s lymphoma: a risk model. Ann Oncol (2002) 13(7):1099–107. 10.1093/annonc/mdf175 12176790

[23] de Graaf MT de Jongste AH Kraan J Boonstra JG Sillevis Smitt PA Gratama JW.Flow cytometric characterization of cerebrospinal fluid cells. Cytometry B Clin Cytom (2011) 80(5):271–81. 10.1002/cyto.b.20603 21567940

[24] Martinez-Laperche C Gómez-García AM Lassaletta Á Moscardó C Vivanco JL Molina J Detection of occult cerebrospinal fluid involvement during maintenance therapy identifies a group of children with acute lymphoblastic leukemia at high risk for relapse. Am J Hematol (2013) 88(5):359–64. 10.1002/ajh.23407 23468276

[25] Ranta S Nilsson F Harila-Saari A Saft L Tani E Söderhäll S Detection of central nervous system involvement in childhood acute lymphoblastic leukemia by cytomorphology and flow cytometry of the cerebrospinal fluid. Pediatr Blood Cancer (2015) 62(6):951–6. 10.1002/pbc.25363 25545289

[26] Sancho JM Orfao A Quijano S García O Panizo C Pérez-Ceballos E Clinical significance of occult cerebrospinal fluid involvement assessed by flow cytometry in non-Hodgkin’s lymphoma patients at high risk of central nervous system disease in the rituximab era. Eur J Haematol (2010) 85(4):321–8. 10.1111/j.1600-0609.2010.01478.x 20528905

[27] de Jongste AH de Graaf MT van den Broek PD Kraan J Sillevis Smitt PA Gratama JW. Effector memory and late memory T cells accumulate in the blood of CMV-carrying individuals but not in their cerebrospinal fluid. Cytometry B Clin Cytom (2013) 84(4):218–21. 10.1002/cyto.b.21073 23401348

